# Integrated quality assessment and pigment profiling in colored rice varieties

**DOI:** 10.1371/journal.pone.0355382

**Published:** 2026-10-09

**Authors:** Shan Yan, Teng Cheng, Changlong Gou, Xingfei Zheng, Lei Wu, Jiawen Li, Nunung Harijati, Deze Xu, Zhongli Hu, Ying Diao, Zhongming Fang

**Affiliations:** 1 School of Life Science and Technology, Wuhan Polytechnic University, Wuhan, Hubei, China; 2 Department of Neurobiology, Key Laboratory of Molecular Neurobiology of the Ministry of Education and the Collaborative Innovation Center for Brain Science, Naval Medical University, Shanghai, China; 3 Institute of Food Crops, Hubei Academy of Agricultural Sciences, Wuhan, Hubei, China; 4 Qianjiang Public Inspection and Testing Center, Qianjiang, Hubei, China; 5 Department of Biology, Faculty of Mathematics and Natural Sciences, University of Brawijaya, Malang, East Java, Indonesia; 6 Hubei Enshi Selenium-rich Resource Observation and Research Station, Enshi, Hubei, China; 7 College of Agricultural Sciences, Guizhou University, Guiyang, Guizhou, China; University of Waterloo, CANADA

## Abstract

Colored rice, available in black, red, purple, and yellow, and green varieties, is recognized for its high nutritional value and potential in modern health-conscious diets. This study systematically evaluated the quality attributes and pigment profiles of diverse colored rice germplasm resources. Principal component analysis revealed that pasting properties, total phenolic content, total flavonoid content, antioxidant capacity, and sensory characteristics were the primary determinants of quality across all colored rice accessions. Furthermore, comprehensive metabolic profiling using UHPLC-MS/MS identified 818 metabolites across 20 pigment extracts, including flavonoids, anthocyanins, phenolic acids, alkaloids, terpenes, and organic heterocyclic compounds. Notably, 11 anthocyanins—predominantly cyanidin-3-glucoside, cyanidin-3-galactoside, cyanidin-3-rutinoside, and malvidin-3-glucoside—along with 3 proanthocyanidins, were identified as the major bioactive components. These findings provide a robust scientific basis for the functional characterization and nutritional evaluation of colored rice, facilitating the future conservation, genetic improvement, and industrial utilization of these valuable germplasm resources.

## Introduction

Rice (*Oryza sativa* L.) is a staple food for billions worldwide and remains the most widely consumed grain in Asia. Among its diverse varieties, colored rice stands out as a specialty crop, recognized for its rich pigmentation—primarily due to the accumulation of anthocyanins and proanthocyanidins in the bran layer, which impart distinctive hues of black, purple, and red [1]. Typically consumed as whole grain brown rice, colored rice retains the bran, germ, and endosperm, thereby offering a superior nutritional profile compared to ordinary milled white rice. In countries like China, Japan, and South Korea, colored rice is highly valued for its unique flavor and its traditional role as a dietary and medicinal food [2]. Research has demonstrated that colored rice is rich in essential amino acids, trace elements, minerals, and polyphenolic compounds (e.g., phenolic acids, flavonoids, and anthocyanins). Consequently, it exhibits significant pharmacological properties, including antioxidant, anti-atherosclerotic, anti-cancer, neuroprotective, anti-obesity, and anti-aging effects [3–10]. The antioxidant capacity of colored rice is predominantly determined by its specific polyphenolic composition [11,12]. These bioactive components are highly concentrated in the bran layer; for instance, in black rice, the bran accounts for approximately 97% of the total anthocyanins, while the endosperm is entirely devoid of them [13]. Despite these nutritional advantages, the widespread adoption of colored rice is hindered by agricultural and sensory limitations, such as grain shattering, low germination rates, and poor eating quality [14]. Although novel germplasms like “Purple Endosperm Rice” [15], “Astaxanthin Rice (aSTARice)” [16], and shorter, early-maturing black rice [17] have been developed, limitations in balancing yield and palatability remain unresolved.

The functional value of colored rice is intrinsically linked to its natural pigment composition in the seed coat. Anthocyanins, the predominant pigments, typically exist conjugated with polysaccharides via glycosidic bonds. While various extraction techniques—ranging from standard solvent maceration using optimized ethanol solutions to ultrasonic and microwave-assisted methods—have been well-documented for isolating these valuable compounds [18,19], systematic metabolic profiling across diverse germplasms is still necessary. Understanding the precise pigment and metabolite composition is an essential prerequisite for evaluating the functional quality of different colored rice resources.

Currently, comprehensive quality evaluation systems for common white rice are well established, providing valuable guidance for high-yield and high-quality breeding. However, despite the growing interest in colored rice, the extensive diversity and broad distribution of its germplasm resources have increased the complexity of modern breeding efforts. To address this knowledge gap, the core objective of this study is to identify the key indicators that determine the balance between the nutritional value and eating quality of colored rice. To achieve this, we systematically evaluated representative colored rice germplasm resources. Specifically, principal component analysis (PCA) was first employed to investigate the underlying correlations among multidimensional indicators, including physicochemical properties, pasting properties, eating quality, total phenolic and flavonoid contents, and antioxidant activity. This analysis aimed to screen for germplasm resources with optimal comprehensive quality and to identify the key factors determining colored rice quality. Concurrently, ultra-high-performance liquid chromatography-tandem mass spectrometry (UHPLC-MS/MS) was utilized to systematically characterize the pigment composition of rice with distinct colors. The findings of this integrated study will provide a scientific basis for establishing comprehensive quality evaluation standards, thereby facilitating the targeted breeding of elite colored rice varieties.

## Methods and materials

### Study design and germplasm collection

To ensure the representativeness of the colored rice germplasm and minimize sampling bias, all rice varieties used in this study were cultivated under identical geographical and agronomic conditions. A total of 71 colored rice germplasm samples were collected from the rice germplasm repository of the Hubei Academy of Agricultural Sciences Institute of Food Crops for this study, consisting of 21 black rice, 24 red rice, 22 purple rice, 3 yellow rice, and 1 green rice. The purple rice group further included 11 purple, 2 purple-red, and 9 medium-purple. Detailed information on these samples can be found in S1 Table, while the different colored rice varieties are visually presented in Fig 1. The rice in this experiment is whole dehusked colored rice.

**Fig 1:**
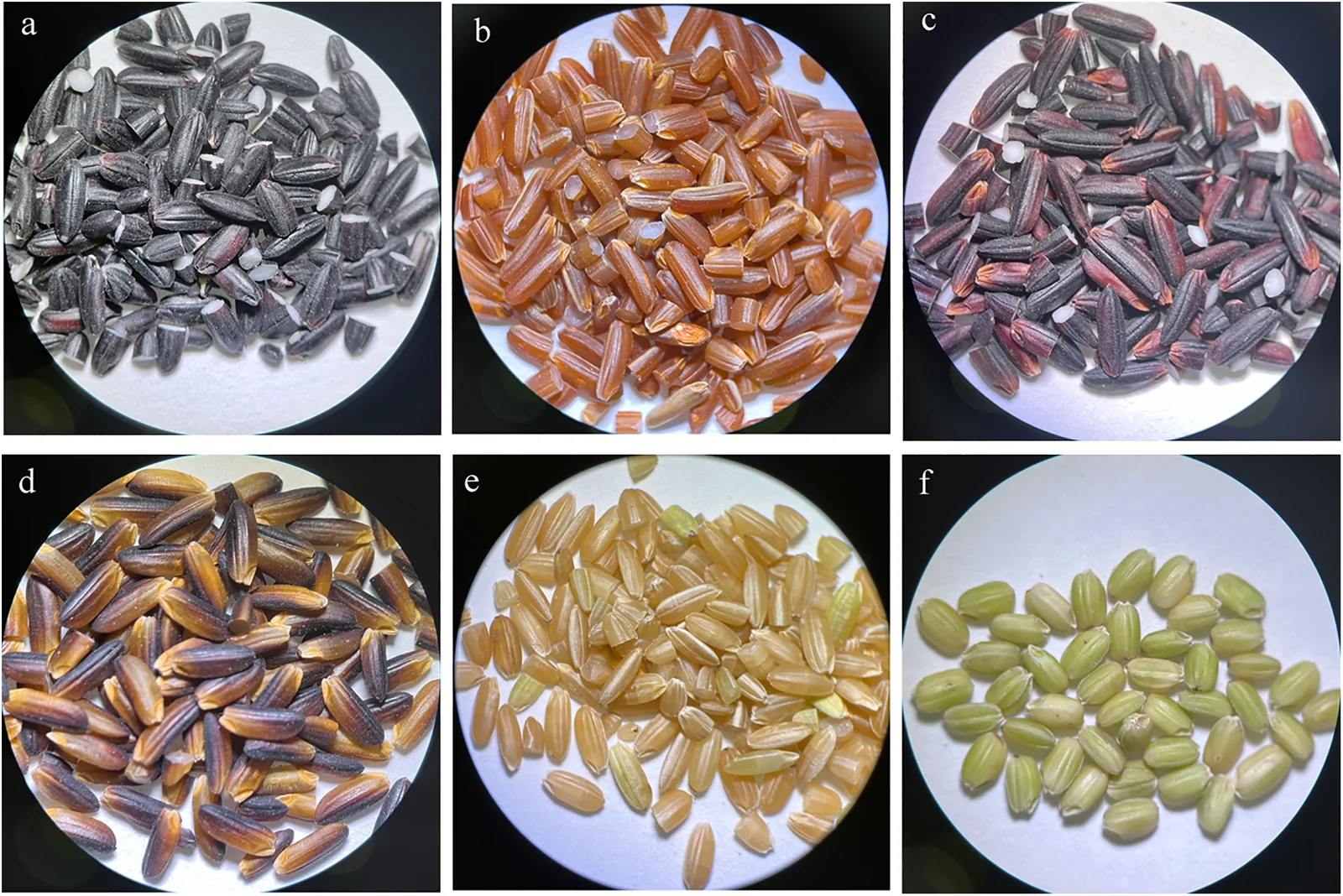
Rice examples of different colored rice materials. Note: Black rice (a); Red rice (b); Purple rice (c); Medium-purple rice (d); Yellow rice (e); Green rice (f).

### Measurement of comprehensive quality indicators

The thousand-grain weight of colored rice was measured by weighing 1,000 grains three times, with the average value used for the final determination. To calculate the length-to-width ratio, five intact grains were randomly selected, and their length and width were measured using a vernier caliper. The moisture content of colored rice was determined according to the direct drying method described in GB/T 5009.3−1985. Crude fiber content was measured following the protocol outlined in GB/T 5009.10−2003, while resistant starch content was assessed according to T/CI 037−2022. The pasting properties of colored rice were evaluated using the method specified in the industry standard GB/T 24852−2010. Fat content was calculated via the Soxhlet extraction method, and soluble protein content was determined using the Coomassie Brilliant Blue assay. The sensory qualities of cooked rice, including taste, viscosity, hardness, balance, and elasticity, were assessed following the guidelines in the Rice Flavor Taste Test Manual. The contents of amylose and amylopectin were quantified based on the method of He et al. [20].

### Extraction of pigments from colored rice

Pigments from colored rice were extracted following a modified method based on Tai et al. [21], combined with the optimal extraction conditions previously optimized by response surface methodology in our laboratory. Briefly, 1 g of colored rice powder (passed through a 100-mesh sieve) was accurately weighed and extracted using 35 mL of extraction solvent, consisting of 65% ethanol and 0.1 M hydrochloric acid in an 85:15 (v:v) ratio. The extraction was carried out at 90 °C for 56 min. After extraction, the dispersion was centrifuged at 3665 × g for 20 min, and the supernatant was carefully separated and extracted in triplicate. The pigment extracts were then concentrated using rotary evaporation and subsequently freeze-dried into powder. The lyophilized pigment powder was stored at −20 °C until further analysis. These extracts were later used to determine total phenolic content, total flavonoid content, antioxidant activity, and for UHPLC-MS/MS analysis.

### Determination of the total phenolic content and total flavonoid content

The total phenolic content (TPC) was determined using a modified Folin-Ciocalteu colorimetric method [22]. A standard solution of gallic acid was prepared at a concentration of 10 mg/mL, and a series of dilutions (0.01, 0.02, 0.04, 0.06, 0.08, 0.1 mg/mL) was used for calibration. For the samples, a working solution of colored rice extract was prepared by diluting the extract to a concentration of 1 mg/mL with methanol. A volume of 0.5 mL of the sample solution was mixed with 2.25 mL of 10% Folin-Ciocalteu reagent. After incubating for 5 min, 2.25 mL of 6% sodium carbonate was added, and the dispersion was vortexed and incubated at 25 °C in the dark for 90 min. The absorbance was then measured at 750 nm, determined in triplicate. The total phenolic content was expressed as gallic acid equivalents (GAE) per 100 g of colored rice sample.

The total flavonoid content (TFC) was determined using the colorimetric method described by Dewanto et al. [23], with quercetin used as a standard. A stock solution of quercetin (1 mg/mL) was prepared, and a series of dilutions (0.02, 0.04, 0.08, 0.16, 0.32 mg/mL) was used for the calibration curve. For the samples, a working solution of colored rice extract was prepared by diluting the extract to 1 mg/mL with methanol. A 0.5 mL aliquot of the sample solution was mixed with 2.25 mL of distilled water, followed by the addition of 0.15 mL of 5% sodium nitrite. The dispersion was allowed to stand for 6 min before adding 0.15 mL of 10% aluminum chloride. After standing for 5 min, 1 mL of 1 M sodium hydroxide was added, and the dispersion was vortexed thoroughly. The absorbance was measured at 510 nm, determined in triplicate. The total flavonoid content was expressed as quercetin equivalents (QE) per 100 g of colored rice sample.

### Antioxidant capacity assessment

#### DPPH radical scavenging activity assay.

The antioxidant activity of the colored rice extracts was evaluated using the DPPH radical scavenging assay, as described by Huang et al. [24]. A stock solution of the colored rice extract was prepared by diluting it to 1 mg/mL with methanol. The DPPH solution (1 mM) was prepared using absolute methanol and stored in the dark to prevent degradation. To perform the assay, 0.5 mL of the 1 mg/mL pigment extract solution was mixed with 2.5 mL of the 0.2 mM DPPH-methanol solution. The dispersion was incubated for 30 min in the dark at room temperature. After incubation, the absorbance was measured at 517 nm, with water serving as the blank control, determined in triplicate. Methanol solution was used as the blank for measuring the DPPH radical scavenging capacity. The calculation formula was as follows:


$$\begin{array}{c} \begin{array}{c} \text{DPPH radical scavenging activity}(\%)=\left[\frac{\left[{\text{A}}_{\text{0}}-{\text{A}}_{\text{1}}\right]}{{\text{A}}_{\text{0}}}\right]\times \text{100}\% \end{array} \end{array}$$

where A_1_ was the absorption of the extract sample, A_0_ was the absorption of blank control sample.

#### ABTS^+^ radical scavenging activity assay.

The antioxidant activity against ABTS^+^ radical was determined using the method described by Chen et al. [25]. Initially, the ABTS^+^ solution was prepared by mixing the ABTS^+^ reagent with potassium persulfate in a 1:1 ratio and allowing it to stand in the dark for 12–16 h. Subsequently, 9.5 mL of the ABTS^+^ solution was combined with 0.5 mL of the pigment extract (1 mg/mL) in a 10 mL centrifuge tube. After a 6 min incubation period, the absorbance was measured at 734 nm, with water serving as the blank control, determined in triplicate. A methanol solution was used simultaneously as the blank for measuring the ABTS^+^ radical scavenging capacity. The calculation formula was as follows:


$$\begin{array}{c} \begin{array}{c} {\text{ABTS}}^{+}\text{ radical scavenging ability}(\%)=\left[\frac{{\text{A}}_{\text{0}}-{\text{A}}_{\text{1}}}{{\text{A}}_{\text{0}}}\right]\times \text{100}\% \end{array} \end{array}$$

where A_1_ was the absorption of the extract sample, A_0_ was the absorption of the blank control sample.

#### Hydroxyl radical scavenging ability assay.

The hydroxyl radical antioxidant activity was determined using the method described by Chen et al. [25]. First, 0.5 mL of 9 mM FeSO_4_ solution and 0.5 mL of 6 mM salicylic acid (dissolved in anhydrous ethanol) were added to 0.5 mL of the pigment extract (1 mg/mL). Following this, 0.5 mL of 8.8 mM H_2_O_2_ was introduced, and the dispersion was thoroughly mixed before being incubated at 37 °C for 30 min. The absorbance of the solution was then measured at 510 nm, with water serving as the blank control, and determined in triplicate. A methanol solution was also used as a blank to simultaneously measure the hydroxyl radical scavenging capacity. The calculation formula was as follows:


$$\begin{array}{c} \begin{array}{c} \text{Hydroxyl radical scavenging ability}(\%)=\left[\frac{{\text{A}}_{\text{0}}-{\text{A}}_{\text{1}}}{{\text{A}}_{\text{0}}}\right]\times \text{100}\% \end{array} \end{array}$$

where A_1_ was the absorption of the extract sample, A_0_ was the absorption of the blank control sample.

#### Ferric reducing antioxidant power assay.

The ferric reducing antioxidant power (FRAP) of the pigment extracts was determined according to the method described by Chen et al. [25], with minor modifications. The FRAP reagent was prepared by mixing 10 mM TPTZ solution (dissolved in 40 mM hydrochloric acid), 300 mM sodium acetate buffer (pH 3.6), and 20 mM ferric chloride in a 1:1:10 ratio. In a 10 mL centrifuge tube, 0.5 mL of the pigment extract (1 mg/mL) was combined with 9.5 mL of the FRAP reagent. The dispersion was thoroughly mixed and allowed to stand for 4 min. Subsequently, the absorbance was recorded at 593 nm, with water serving as the blank control, determined in triplicate. A methanol solution was also used as a blank to measure the FRAP reducing capacity simultaneously. The calculation formula was as follows:


$$\begin{array}{c} \begin{array}{c} \text{FRAP reducing activity}(\%)=\left(\frac{{\text{A}}_{\text{1}}-{\text{A}}_{\text{0}}}{{\text{A}}_{\text{0}}}\right)\times \text{100}\% \end{array} \end{array}$$

where A_1_ was the absorption of the extract sample, A_0_ was the absorption of the blank control sample.

### UHPLC-MS/MS analysis

For metabolite extraction, 5 mg of the sample (d.b.) was weighed and combined with 1000 µL of the extraction solution, consisting of methanol, acetonitrile, and water in a 2:2:1 (v:v:v) ratio. The dispersion was ground at 35 Hz for 4 min, followed by sonication in an ice-water bath for 5 min. This grinding and sonication process was repeated three times. The sample was then allowed to stand at −40 °C for 1 h, after which it was centrifuged at 4 °C at 2,4149 × g for 15 min. The supernatant was collected into a sample vial for further analysis. UHPLC-MS/MS analyses were conducted using a Vanquish UHPLC system (Thermo Fisher Scientific) equipped with a Phenomenex Kinetex C18 column (2.1 mm × 50 mm, 2.6 μm) and coupled to an Orbitrap Exploris 120 mass spectrometer (Thermo). The mobile phase consisted of 0.01% acetic acid in water as phase A, and a dispersion of isopropanol and acetonitrile (1:1, v:v) as phase B. The auto-sampler was maintained at 4 °C, and the injection volume was set at 2 μL. The Orbitrap Exploris 120 mass spectrometer was operated in information-dependent acquisition (IDA) mode, controlled by the Xcalibur acquisition software. This software continuously evaluates the full scan MS spectrum during analysis. The ESI source conditions were optimized as follows: sheath gas flow rate was set to 50 Arb, aux gas flow rate to 15 Arb, and capillary temperature to 320 °C. The full MS resolution was set to 60,000, and the MS/MS resolution to 15,000. Collision energy parameters were defined as SNCE 20/30/40, and the spray voltage was maintained at 3.8 kV for positive ionization and −3.4 kV for negative ionization.

### Statistical analysis

The quality data of colored rice were analyzed using Microsoft Excel to determine the coefficient of variation. Pearson correlation analysis and PCA were conducted using SPSS 23 to evaluate the quality variations among colored rice accessions. To further validate the reliability of the PCA results, hierarchical clustering analysis (HCA) was performed; we utilized the median linkage method for clustering and Euclidean distance as the similarity measure, following column-wise Min-Max standardization of the quality indicators. For metabolomics data, raw files were converted to mzXML format utilizing ProteoWizard. Subsequent processing, including peak detection, extraction, alignment, and integration, was performed using an in-house program developed in *R* based on the XCMS framework. Metabolite annotation was carried out against an in-house MS2 database (BiotreeDB), with a similarity cutoff threshold of 0.3. To ensure data reliability, all metabolomic and quality assays were performed with three biological replicates and two technical replicates. Data anomalies and outliers were rigorously evaluated and excluded using Grubbs’ test (*p* < 0.05). Furthermore, the criteria for metabolite identification were strictly defined, requiring a mass error of < 5 ppm and an MS/MS match score > 0.8.

## Results and discussion

### Quality analysis of colored rice

#### Analysis of the basic nutritional quality of colored rice.

The basic nutritional and physicochemical attributes of the colored rice resources, including appearance, taste, and antioxidant capacity, are summarized in Table 1 and S2 Table. Morphological traits, such as thousand-grain weight, grain length, grain width, and length-width ratio, exhibit relatively low coefficients of variation (CV), indicating morphological stability across the germplasm. Specifically, the length-width ratio (CV: 17.92%) ranges from the slender RR5 (3.8) to the short-round GR1 (1.45). Red rice accessions are generally slender (mean 3.13), whereas yellow (mean 2.11) and green (1.45) rice accessions are relatively short and round. In contrast, biochemical composition exhibits substantial genetic diversity. Resistant starch content shows high variability (CV: 59.31%), ranging from 0.29% in PR12 to 3.05% in YR3; the ranges for black, red, and purple rice are 0.3–1.09%, 0.42–1.97%, and 0.29–1.14%, respectively. Crude fiber and amylose contents display CVs of 45.03% and 40.34%, respectively, with red rice accessions showing the greatest heterogeneity in crude fiber (0.11–0.93%). Amylose content peaks in RR7 (32.04%) and reaches its minimum in GR1 (4.77%). Further variability is observed in amylopectin (CV: 14.11%) and soluble protein (CV: 18.19%) across all accessions. These findings are consistent with previous reports by Ye et al. [26] and Sui et al. [27], which noted that while total starch content remains relatively stable among brown rice germplasm, significant variations exist in protein and amylose profiles. Such biochemical diversity is critical for industrial applications: high-amylose accessions are ideal for products requiring structural integrity, such as extruded snacks and noodles, whereas low-amylose accessions are preferred for soft, moist food applications where tenderness and moisture retention are prioritized [28].

**Table 1:** Coefficient of variation of colored rice resources.

Index	71 Colored rice				21 Black rice				24 Red rice				22 Purple rice			
	Min	Max	Mean	CV (%)	Min	Max	Mean	CV (%)	Min	Max	Mean	CV (%)	Min	Max	Mean	CV (%)
Thousand-grain weight (g)	14.76	26.78	19.84	12.95	16.81	24.29	19.87	10.75	14.76	26.78	20.37	15.35	14.95	23.28	19.46	11.31
Grain length (cm)	0.43	0.77	0.66	9.9	0.51	0.74	0.63	8.46	0.58	0.77	0.69	7.39	0.58	0.76	0.68	6.43
Grain width (cm)	0.19	0.31	0.24	11.46	0.21	0.31	0.25	9.61	0.20	0.27	0.22	8.41	0.19	0.28	0.23	10.71
Length-width ratio	1.42	3.83	2.85	17.92	1.67	3.09	2.54	15.11	2.31	3.83	3.13	11.84	2.17	3.68	3.00	14.10
Water content (%)	9.8	15.47	11.42	12.16	10.00	15.47	11.88	15.28	9.80	13.00	11.32	8.44	9.93	14.20	10.97	11.52
Fat content (%)	2	9.08	5.77	22.8	3.50	9.08	6.08	24.44	3.25	7.33	5.41	19.80	4.08	8.42	5.96	19.61
Soluble protein content (mg/g)	1.48	3.99	3.06	18.19	2.23	3.67	3.13	13.51	1.48	3.99	2.80	25.98	2.18	3.87	3.27	11.46
Crude fiber content (%)	0.11	0.93	0.34	45.03	0.18	0.87	0.37	38.15	0.11	0.93	0.30	60.71	0.16	0.73	0.34	39.34
Resistant starch content (%)	0.29	3.05	0.71	59.31	0.30	1.09	0.58	41.58	0.42	1.97	0.82	48.44	0.29	1.14	0.62	33.72
Amylose content (%)	4.77	32.04	13.88	40.34	5.14	18.01	11.67	30.53	6.31	32.04	15.66	44.36	5.27	21.10	13.63	26.60
Amylopectin content (%)	38.58	67.93	52.88	14.11	41.22	64.91	51.60	13.07	38.58	67.90	55.52	13.67	39.50	65.96	50.79	12.06
Taste values	30	53.7	32	14.77	30.00	53.70	34.57	19.31	30.00	30.00	30.00	0.00	30.00	47.80	31.96	14.09
Appearance	0.2	4	0.27	173.54	0.20	4.00	0.42	198.81	0.20	0.20	0.20	0.00	0.20	0.20	0.20	0.00
Mouthfeel	0.2	4.4	0.63	145.06	0.20	4.40	1.15	110.09	0.20	0.30	0.20	10.00	0.20	3.40	0.62	139.39
Hardness	0.3	2.73	1.14	49.01	0.30	1.96	0.92	52.85	0.63	2.73	1.55	31.93	0.31	1.86	0.92	49.00
Viscosity	0.03	0.33	0.08	52.01	0.03	0.13	0.07	34.48	0.03	0.18	0.07	45.75	0.05	0.33	0.10	56.49
Balance	0.01	0.25	0.09	67.87	0.02	0.21	0.11	58.86	0.01	0.17	0.05	67.84	0.05	0.25	0.12	53.67
Elasticity	0.88	0.99	0.94	2.05	0.88	0.96	0.94	2.18	0.92	0.98	0.95	1.27	0.88	0.99	0.94	2.51
PKV (cP)	280	2957.33	1704.38	32.64	280.00	1982.67	1358.63	29.55	1329.00	2957.33	2157.36	16.08	601.33	2554.00	1605.86	33.34
HPV (cP)	167.33	1735	941.56	45.72	167.33	1084.00	644.40	40.78	835.00	1735.00	1338.94	15.06	195.33	1523.67	830.77	50.38
CPV (cP)	259.33	3335.33	1800.31	55.25	259.33	2216.00	1100.05	53.51	2158.67	3335.33	2703.42	12.07	282.33	3052.33	1510.57	66.71
BDV (cP)	55	1286.67	767.53	36.28	112.67	1165.33	714.27	41.61	470.00	1222.33	832.31	23.67	55.00	1286.67	775.11	43.51
CSV (cP)	81.33	2057.33	859.34	72.14	88.00	1363.00	455.65	94.66	823.33	2057.33	1364.81	21.42	87.00	1655.00	681.34	91.60
SBV (cP)	−1135.67	1440	103.45	641.36	−1036.33	590.33	−258.59	202.91	−298.67	1440.00	546.06	76.29	−1135.67	1052.00	71.05	1020.03
PeT (min)	3.42	6.38	5.29	18.85	3.42	6.16	4.83	19.44	5.58	6.27	6.00	2.90	3.67	6.38	5.02	22.34
PaT (℃)	69.88	92.27	83.61	8.29	69.88	92.27	81.63	8.98	81.55	90.98	88.09	2.76	70.22	90.73	81.12	9.21
Pigment extract (mg/g)	41.37	82.8	58.36	15.2	47.40	76.40	59.00	13.51	42.90	82.47	57.57	16.97	41.37	82.80	59.55	15.76
TPC (mg GAE/100g)	200.22	1166.13	658.1	36.51	474.43	1166.13	795.50	26.43	218.03	1022.62	635.28	28.40	232.58	1098.45	629.09	38.83
TFC (mg QE/100g)	180.4	5007.53	2396.58	45.4	1393.98	5007.53	2945.53	35.47	869.78	4230.71	2525.39	29.44	399.18	4691.05	2104.26	49.61
DPPH radicals scavenging capacity (%)	31.22	86.46	63.52	18.51	50.26	86.46	69.34	14.66	41.13	79.43	65.03	15.80	44.22	75.94	60.16	16.66
ABTS^+^ radicals scavenging capacity (%)	13.7	91.51	53.03	36.78	39.94	88.40	64.30	22.47	22.84	91.51	55.81	30.37	21.05	81.07	45.86	38.48
Hydroxyl radical scavenging capacity (%)	2.37	16.76	8.65	32.98	5.92	16.76	10.90	25.96	2.37	10.94	6.62	32.88	5.96	12.46	9.07	20.53
FRAP radicals reducing capacity (%)	35.9	381.07	173.43	47.8	124.12	381.07	218.00	33.74	47.96	346.4	173.02	43.70	74.42	318.62	154.97	49.66

Note: “CV” indicates coefficient of variation.

#### Analysis of the eating quality of colored rice.

The cooking and eating quality of the 71 colored rice accessions exhibited significant diversity. Black rice achieved the highest mean taste score (34.57), followed by purple (31.96) and red rice (30.00). Across all samples, taste values ranged from 30.0 to 53.7, yielding an overall CV of 14.77%. In terms of appearance, black rice displayed extreme variability (CV: 198.81%), whereas the red and purple accessions remained highly uniform. Textural analysis revealed a mean hardness of 1.14 across the 71 accessions; red rice exhibited the highest hardness (mean 1.55), while black and purple rice were relatively softer (mean 0.92 for both). Furthermore, the three yellow rice accessions and one green rice accession (GR1) evaluated in this study all recorded a baseline taste value of 30.0, yet they demonstrated distinct textural properties. Yellow rice exhibited greater hardness (1.172) and a lower mouthfeel score (0.467) compared to green rice, which presented lower hardness (0.62) and a relatively superior mouthfeel (0.60). Overall, the taste values of the colored brown rice evaluated in our study are fundamentally consistent with those reported by Ren et al. [29], who documented a range of 30.0 to 48.5.

The taste quality of cooked rice is a complex trait primarily governed by protein and amylose content. Amylose content demonstrated a highly significant positive correlation with rice hardness; accessions with elevated amylose levels tend to produce harder-cooked rice that is susceptible to retrogradation, thereby diminishing overall taste quality. Similarly, higher protein levels in brown rice contribute to a firmer cooked texture and a corresponding reduction in taste scores [30]. Physical grain dimensions also play a critical role: grain thickness is extremely positively correlated with cooked rice taste value, with an average increase of 1.5 points observed for every 0.1 mm increase in thickness [31]. In contrast, grain length demonstrates no significant correlation with taste quality [32]. Finally, the intact bran layer in brown rice provides essential structural integrity, yielding a firmer mouthfeel while simultaneously slowing starch digestion and promoting satiety. Because bioactive compounds, such as phenolics and flavonoids, are predominantly concentrated in the bran layer, colored rice accessions retaining this structure hold significant potential as functional dietary staples capable of enhancing satiety and modulating metabolic health.

#### Analysis of the pasting properties of colored rice.

Significant genetic variations in pasting properties were observed across the diverse colored rice accessions. Among the entire set of 71 colored rice accessions, the overall mean values for peak viscosity (PKV), hot paste viscosity (HPV), cool paste viscosity (CPV), breakdown viscosity (BDV), consistency viscosity (CSV), and setback viscosity (SBV) were 1704.38 cP, 941.56 cP, 1800.31 cP, 767.53 cP, 859.34 cP, and 103.45 cP, respectively. The corresponding CVs were 32.64%, 45.72%, 55.25%, 36.28%, 72.14%, and an exceptionally high 641.36%. The extreme variability in SBV (ranging from −1135.67 to 1440.00 cP) highlights profound phenotypic divergence in retrogradation tendencies across the germplasm.

Among the color subgroups, red rice exhibited the highest mean values for PKV, HPV, CPV, BDV, CSV, and SBV, while maintaining relatively low intra-group CVs of 16.08%, 15.06%, 12.07%, 23.67%, 21.42%, and 76.29%, respectively. In contrast, purple rice displayed extreme intra-group variability in SBV (CV: 1020.03%), and black rice also exhibited a substantial CV of 202.91% for SBV. Additionally, the CVs for CSV in black and purple rice were considerably high at 94.66% and 91.60%, respectively. Furthermore, the pasting profiles of yellow and green rice were quantified: yellow rice demonstrated moderate to high viscosity parameters (e.g., PKV: 1524.00 cP; CPV: 2103.78 cP; SBV: 579.78 cP). Conversely, green rice exhibited substantially lower pasting profiles across all evaluated parameters (PKV: 802.00 cP; CPV: 295.00 cP; SBV: −506.67 cP).

These findings align with the results of Ye et al. [26], who reported significant inter-varietal differences (CV > 15%) in HPV, CPV, BDV, and SBV among 24 brown rice accessions. In the present study, the elevated CPV, PKV, and SBV observed in red rice are consistent with its relatively high amylose content. Mechanistically, lipids and proteins present in the bran layer of colored rice can form amylose-lipid or amylose-protein complexes that restrict water absorption during cooking and inhibit starch swelling. This interaction typically leads to decreased PKV and BDV, alongside increased SBV and CPV [33], ultimately impairing the eating quality of the cooked rice. Elevated PKV and SBV are generally indicative of higher amylose content, a pattern distinctly observed in red rice compared to black and purple rice [34]. Therefore, breeding strategies aimed at enhancing the palatability of colored rice should prioritize accessions with lower amylose content and strictly integrate the systematic evaluation of pasting properties into the selection framework.

#### Analysis of TPC, TFC, and antioxidant activity of colored rice.

Significant phenotypic variations in pigment, TPC, TFC, and antioxidant capacities were observed across the 71 colored rice accessions. Within the entire germplasm collection, the overall mean pigment content was 58.36 mg/g (CV: 15.20%). Phenolic and flavonoid compounds, which represent the principal bioactive constituents, exhibited profound genetic diversity across the 71 accessions: the overall mean TPC and TFC were 658.10 mg GAE/100 g and 2396.58 mg QE/100 g, with remarkably high CV of 36.51% and 45.40%, respectively (S2 Table).

The accumulation of these bioactive compounds strongly correlates with hull color. Current literature generally suggests that darker-colored rice accessions accumulate higher levels of flavonoids [35]. While previous studies present conflicting reports regarding whether black or red rice possesses higher phenolic and flavonoid contents [34,36], our comprehensive dataset definitively establishes the superiority of black rice. Specifically, the mean TPC in black rice (795.50 mg GAE/100 g) significantly exceeded that of red (635.28 mg GAE/100 g) and purple rice (629.09 mg GAE/100 g). Similarly, the TFC in black rice (2945.53 mg QE/100 g) was substantially higher than in red (2525.39 mg QE/100 g) and purple rice (2104.26 mg QE/100 g). Among the lighter-colored accessions, yellow rice exhibited higher TPC and TFC (244.14 mg GAE/100 g and 363.48 mg QE/100 g, respectively) compared to the green rice accession (GR1) (200.57 mg GAE/100 g and 307.94 mg QE/100 g).

Consistent with the accumulation patterns of these bioactive compounds, the antioxidant capacities across the 71 accessions displayed broad phenotypic variation. The overall mean values for DPPH, ABTS^+^, and hydroxyl radical scavenging capacities, alongside FRAP reducing power, were 63.52%, 53.03%, 8.65%, and 173.43%, respectively, with CVs ranging broadly from 18.51% to 47.80%. In comparative subgroup analyses, black rice demonstrated superior antioxidant performance over red rice across all evaluated assays, exhibiting higher DPPH (69.34% vs. 65.03%), hydroxyl (10.90% vs. 6.62%), and ABTS^+^ (64.30% vs. 55.81%) radical scavenging activities, as well as greater FRAP reducing power (218.00% vs. 173.02%). When comparing yellow and green rice, yellow rice displayed stronger DPPH, hydroxyl radical scavenging capacities, and FRAP reducing power, although its ABTS^+^ scavenging capacity was slightly lower than that of green rice (16.45% vs. 17.14%). Overall, these findings strongly corroborate the hypothesis proposed by Qin et al [37], asserting that flavonoids are the primary polyphenolic drivers of antioxidant activity in rice, with phenolic compounds accounting for the vast majority of the antioxidant capacity observed in brown rice [38].

### Correlation analysis

To comprehensively investigate the interplay between various quality indicators, a Pearson correlation analysis of 33 parameters was conducted across all 71 colored rice accessions using Origin 2022 (Fig 2). This unified analysis revealed the universal physicochemical mechanisms driving colored rice quality.

**Fig 2:**
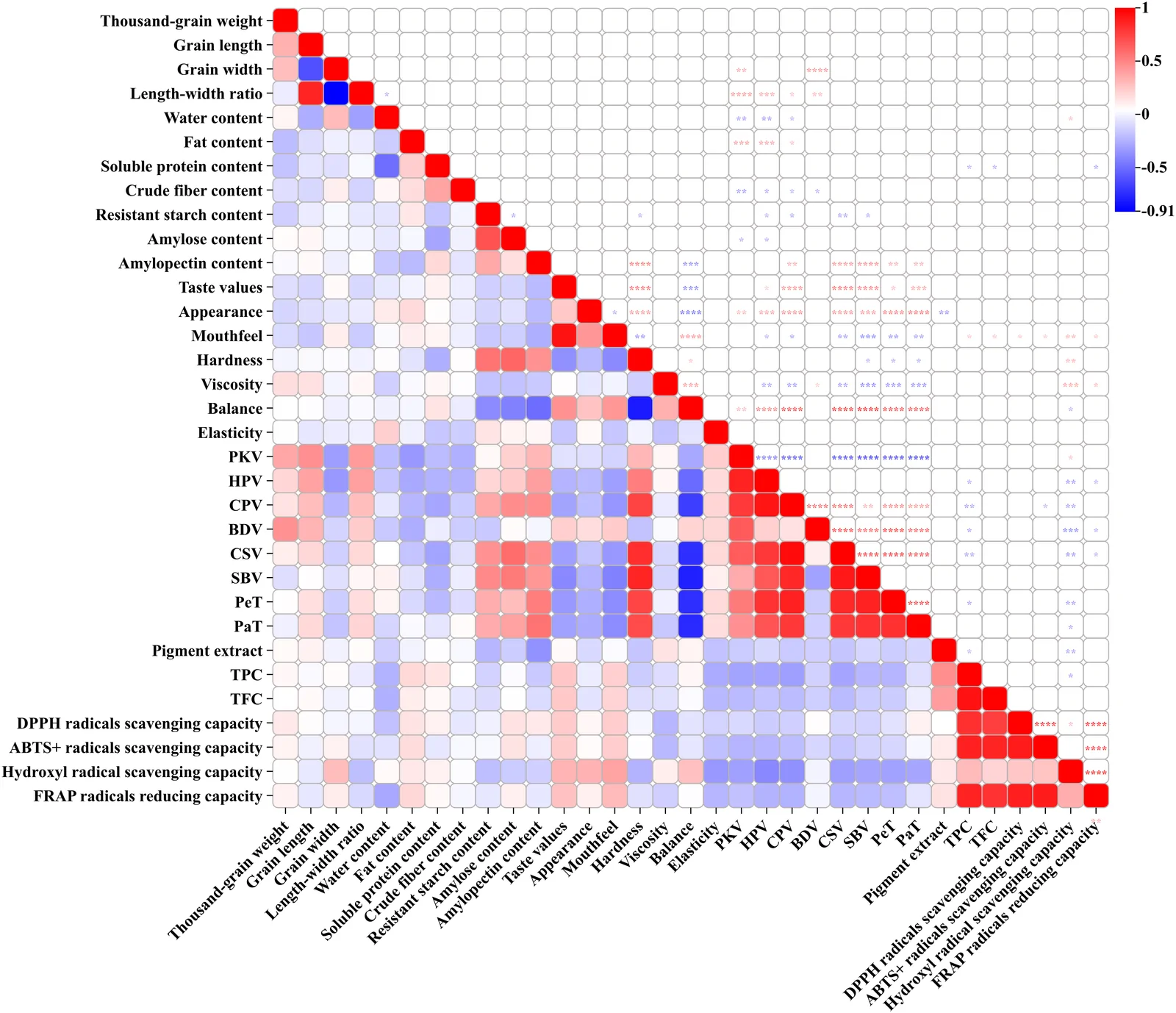
The correlation analysis of the colored rice. Note: * mean *P*≤0.05；** mean *P*≤0.01.

Eating quality parameters (taste value, appearance, mouthfeel, viscosity, and balance) exhibited highly significant positive correlations with one another. However, they were strongly negatively correlated with hardness, amylose content, amylopectin content, and specific pasting properties, including SBV, CSV, PaT, and PeT. Conversely, amylose and amylopectin contents showed significant positive correlations with hardness, CSV, SBV, PeT, and PaT. This indicates that elevated amylose levels result in harder-cooked rice that is highly susceptible to retrogradation (indicated by high SBV), which subsequently impairs the overall taste quality. These relationships collectively indicate that elevated amylose levels increase cooked rice hardness and promote amylose reassociation and retrogradation during cooling (indicated by high SBV and CPV), which ultimately impairs the overall taste quality [29,39].

Beyond biochemical composition, pasting properties are intricately linked to rice quality and serve as effective physicochemical indicators of texture. Generally, larger PKV and BDV values, combined with a lower SBV, indicate superior cooking quality, reflecting minimal retrogradation and texture hardening upon cooling [40,41]. Our current results confirm these dynamics, demonstrating a significant positive correlation between eating quality and BDV, alongside a negative correlation with SBV. Furthermore, the pasting, thermal, and functional properties of colored rice are modulated by the interplay of amylose, lipid, and protein contents [28]. In this study, amylose content showed significant positive correlations with hardness, HPV, PKV, CPV, SBV, CSV, PaT, and PeT. Notably, while some studies have reported a positive correlation between grain length-to-width ratio and eating quality [42], our comprehensive investigation found no significant relationship between physical grain dimensions (length, width, or their ratio) and eating quality, aligning with the observations of Zhu et al. [32].

Regarding antioxidant capacities, the correlation matrix revealed a highly significant positive network linking key bioactive compounds and functional assays. Specifically, TFC showed a strong positive correlation with TPC. Both TPC and TFC were universally and significantly correlated with multiple antioxidant capacity indicators, including DPPH, hydroxyl, and ABTS^+^ radical scavenging abilities, as well as FRAP reducing power. These findings corroborate previous research by Chattopadhyay et al. [30], reinforcing the established reliability of the relationships between phenolic compounds and antioxidant activities in colored rice accessions.

### Comprehensive quality evaluation of colored rice

To comprehensively evaluate the quality of various colored rice cultivars, we performed a PCA in SPSS 22 using 33 physicochemical and sensory quality indices. The suitability of the dataset for factor analysis was assessed using the Kaiser-Meyer-Olkin (KMO) test for sampling adequacy and Bartlett’s test of sphericity. The KMO value was 0.633, and Bartlett’s test of sphericity yielded a *P*-value of less than 0.001, indicating that the variables were sufficiently correlated to perform factor extraction (S3 Table).

Based on the standard criterion of retaining components with an eigenvalue greater than 1 (Eigenvalue > 1), ten principal components (PCs) were extracted for the colored rice samples, accounting for a cumulative contribution rate of 84.86% (S4 Table). The loading coefficients for all 10 PCs are summarized in S5 Table. Fig 3 illustrates the loading and score plots for the first four PCs. PC1 accounted for 27.3% of the total variance, with high positive loadings for pasting properties, including HPV, CPV, CSV, SBV, PeT, PaT, and hardness. Both red and medium-purple rice cultivars exhibited high scores on PC1, reflecting strong gelatinization characteristics. PC2 accounted for 14.2% of the variance and was primarily driven by antioxidant-related indices, including TPC, TFC, and scavenging/reducing capacities (DPPH, ABTS^+^, and FRAP). High scores for this component were observed in several black and purple rice cultivars, indicating superior antioxidant properties (Fig 3a & b). PC3 (10.8%) primarily reflected appearance-related indices, with high positive loadings for grain length, length-width ratio, and BDV; red and purple rice cultivars showed high scores on this axis, corresponding to slender grain shapes. PC4 (7.31%) was mainly associated with physical and sensory properties, including thousand-grain weight, water content, taste, BDV, and mouthfeel (Fig 3c & d). The remaining PCs (PC5–PC10) accounted for 5.76%, 4.87%, 4.46%, 3.76%, 3.36%, and 3.07% of the variance, respectively, representing variables such as overall appearance, soluble protein content, elasticity, and amylose content.

**Fig 3:**
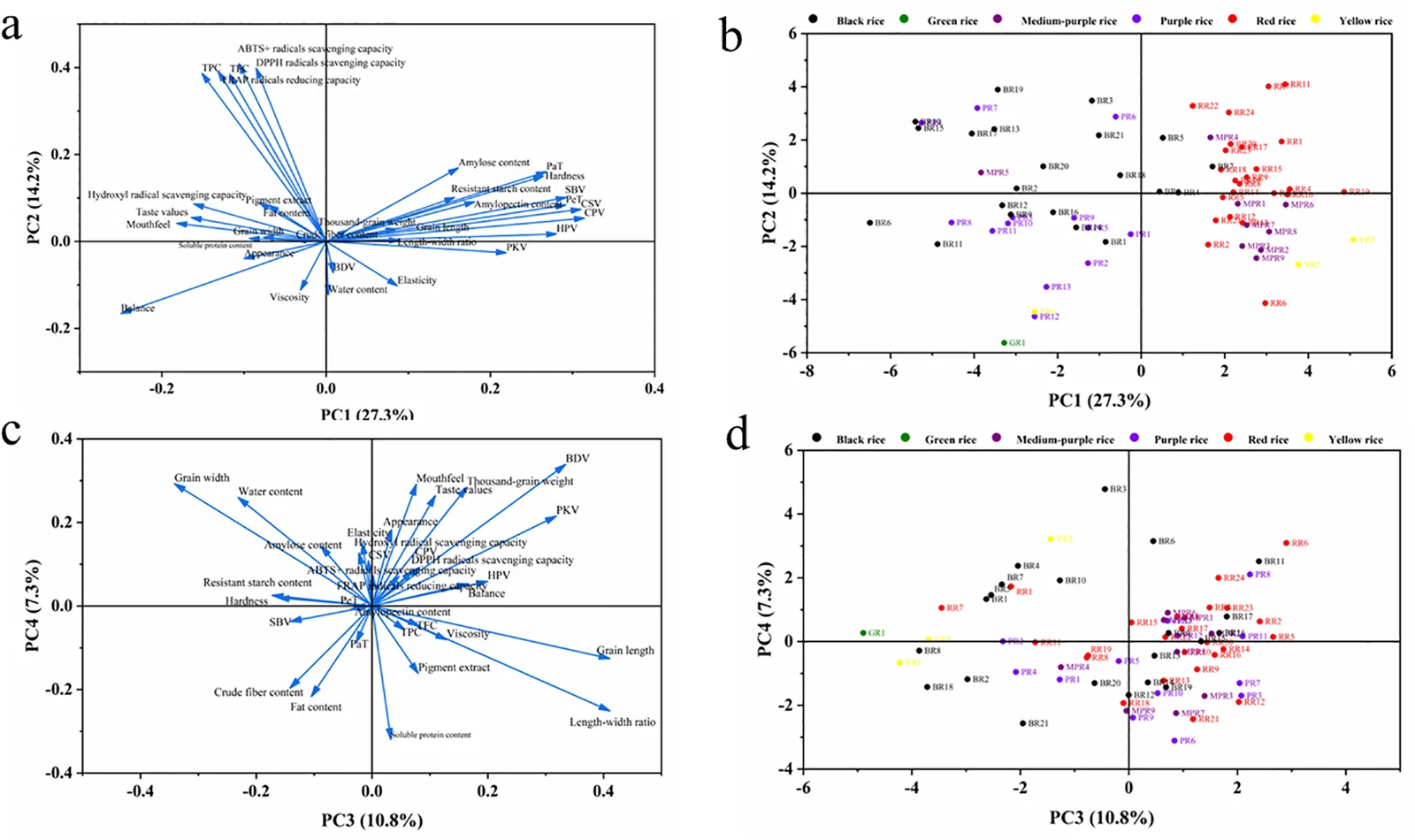
Loading and scores plots of the PCA results of the 33 indicators of colored rice. Note: Loading plot of PC1 and PC2 (a); Scores plot of PC1 and PC2 (b); Loading plot of PC3 and PC4 (c); Scores plot of PC3 and PC4 (c).

Based on the aforementioned PCA results, this study identifies pasting properties, TPC, TFC, and antioxidant capacity as the core dimensions determining the overall quality of colored rice. The high loading scores of these indices within the principal component space reveal an intrinsic link between nutritional functionality and processing characteristics in colored rice. These core indicators are highly consistent with findings from previous studies. For instance. Ren et al. [29] demonstrated that hardness, viscosity, amylose content, and mineral composition are key factors influencing the eating quality of colored brown rice. Concurrently, Qin et al. [37] performed PCA on diverse rice varieties and extracted principal components that accounted for the majority of the variance, further validating the pivotal role of amylose and essential minerals in rice quality classification. In summary, pasting properties, TPC, TFC, and antioxidant capacity constitute the fundamental framework for evaluating colored rice quality. These findings not only provide theoretical support for understanding the phenotypic variation of colored rice but also offer a robust scientific basis for selecting superior breeding materials and establishing standardized quality assessment protocols.

To achieve an objective assessment of the colored rice cultivars, a comprehensive quality evaluation model was established based on PC scores. The linear function for each PC was derived from the eigenvector coefficients (S6 Table). A weighted comprehensive scoring model was then constructed, with weights determined by the proportion of the variance contribution of each PC to the cumulative variance:

Comprehensive~score =0.321×F1+0.167×F2+0.127×F3+0.086×F4+0.068×F5+0.057×F6+0.053 × F7+0.045×F8+0.040×F9+0.036×F10

The comprehensive scores and rankings for all cultivars are summarized in Table 2. Cultivars RR24, RR11, MPR1, RR22, and MPR6 achieved the highest comprehensive scores, representing superior overall quality, whereas GR1 exhibited the lowest rank. This model provides a robust scientific framework for the quantitative quality assessment of diverse rice varieties.

**Table 2:** Comprehensive scores of PCA.

Sample	Comprehensive score	PC1	PC2	PC3	PC4	PC5	PC6	PC7	PC8	PC9	PC10	Ranking
RR24	0.63	0.70	1.40	0.88	1.29	−1.23	−0.43	−0.42	1.21	0.06	0.63	1
RR11	0.53	1.15	1.89	−0.92	−0.01	1.78	−2.32	−0.24	1.50	−1.22	−0.76	2
MPR1	0.51	0.77	−0.18	0.55	0.48	0.70	0.59	0.18	0.52	0.33	1.52	3
RR22	0.51	0.41	1.52	0.36	0.09	−0.92	1.28	−1.10	0.19	1.30	1.52	4
MPR6	0.50	1.15	−0.20	0.38	0.58	0.04	0.80	0.02	0.24	−0.40	0.40	5
RR17	0.48	0.80	0.80	0.52	0.26	0.59	0.40	−1.64	1.72	−0.97	−0.28	6
RR4	0.45	1.19	0.07	0.47	0.51	−0.45	−1.91	0.67	0.35	0.49	0.60	7
RR19	0.45	1.62	0.02	−0.40	−0.28	1.14	−0.18	0.33	−0.56	−0.09	−1.61	8
RR3	0.44	1.06	0.00	0.79	0.69	−1.20	−1.67	1.38	−0.09	0.50	0.73	9
BR3	0.42	−0.39	1.61	−0.24	3.08	1.35	0.68	0.78	−2.09	0.01	−0.94	10
RR1	0.42	1.12	0.90	−1.15	1.11	−1.68	−1.70	2.84	1.34	−1.36	0.54	11
RR23	0.42	0.67	0.75	0.96	0.68	−1.16	0.20	−0.63	−0.59	−0.34	1.03	12
RR20	0.41	0.71	0.86	0.76	−0.01	−0.11	0.12	−0.62	−0.75	0.09	0.12	13
RR18	0.41	0.64	0.41	−0.05	−1.24	1.00	1.57	−0.19	0.33	0.95	1.23	14
MPR3	0.40	0.81	−0.92	0.74	−1.10	−0.50	1.67	2.56	1.48	−0.36	1.17	15
RR7	0.39	1.02	1.86	−1.83	0.68	0.20	−1.59	−0.43	0.97	−1.00	0.57	16
MPR7	0.39	0.84	−0.55	0.46	−1.45	0.78	0.05	1.02	1.66	0.86	1.57	17
RR9	0.37	0.84	0.28	0.67	−0.56	−0.10	0.62	0.25	−0.11	0.33	−0.82	18
MPR8	0.36	1.02	−0.67	0.47	−0.20	0.64	0.28	−0.35	0.35	0.36	0.86	19
RR6	0.35	0.99	−1.91	1.54	1.99	−1.97	0.38	1.03	1.13	0.45	−0.84	20
MPR2	0.31	0.96	−0.99	0.81	0.16	−0.16	1.02	−0.15	−0.03	−0.49	0.82	21
MPR4	0.30	0.55	0.97	−0.67	−0.52	0.29	1.78	0.94	0.45	0.06	−2.81	22
RR12	0.29	0.71	−0.41	1.08	−1.22	−0.25	0.34	0.91	−0.20	1.08	0.50	23
RR14	0.28	0.74	0.02	0.92	−0.15	−0.04	0.00	−0.56	−0.67	0.00	−0.12	24
YR2	0.27	1.25	−1.24	−0.77	2.07	−0.42	1.89	−0.17	−1.14	−0.41	−0.24	25
RR16	0.25	1.17	−0.02	0.84	−0.27	0.42	−0.29	−0.95	−2.60	−0.60	−0.79	26
YR3	0.25	1.69	−0.81	−1.96	0.04	2.39	−0.34	−0.26	2.24	−2.83	−0.92	27
RR10	0.24	0.75	0.22	0.55	−0.21	−0.27	−0.11	−0.73	−0.85	0.74	−0.39	28
RR5	0.23	0.65	−0.07	1.41	0.10	0.11	−1.55	−0.70	−0.48	0.56	−0.92	29
RR15	0.22	0.92	0.42	0.03	0.39	0.21	0.07	−1.38	−1.66	−0.75	−0.66	30
BR5	0.21	0.17	0.96	−1.35	0.94	−0.39	−0.53	0.05	0.66	2.53	0.26	31
RR13	0.16	0.81	−0.52	0.34	−0.79	0.41	0.15	0.12	−0.86	−0.41	0.70	32
RR8	0.16	0.78	0.17	−0.41	−0.32	0.91	0.24	−1.10	−1.66	0.27	0.11	33
RR21	0.15	0.59	−0.47	0.63	−1.57	0.25	0.01	0.41	−0.40	0.79	1.01	34
BR7	0.14	0.57	0.46	−1.24	1.16	−1.06	1.02	−0.31	−0.56	0.33	−0.48	35
MPR9	0.14	0.92	−1.13	−0.02	−1.40	1.54	1.44	1.06	−0.40	0.82	−2.88	36
BR4	0.10	0.30	0.01	−1.09	1.53	0.24	−0.03	−0.39	−0.30	0.21	0.48	37
PR6	0.07	−0.20	1.33	0.45	−2.00	0.08	−0.91	0.27	−0.09	0.65	1.12	38
RR2	0.03	0.53	−0.89	1.28	0.41	−0.18	−1.44	−1.41	−0.90	−0.17	0.74	39
BR8	−0.04	0.15	0.03	−2.05	−0.18	0.27	1.28	−0.59	1.31	1.93	−0.24	40
BR11	−0.06	−1.62	−0.88	1.27	1.62	2.19	1.12	1.94	−0.44	−1.05	1.42	41
BR19	−0.12	−1.14	1.80	0.36	−0.92	−0.02	0.64	0.64	−0.54	−0.36	−1.37	42
BR20	−0.14	−0.78	0.47	−0.34	−0.84	−0.33	1.12	0.34	1.46	1.13	−0.58	43
PR7	−0.14	−1.31	1.48	1.08	−0.84	0.97	−1.09	1.49	−0.81	−1.09	−0.98	44
BR21	−0.15	−0.34	1.01	−1.04	−1.65	0.40	−0.12	0.07	−0.40	0.36	1.25	45
PR8	−0.18	−1.51	−0.51	1.18	1.36	1.48	0.35	0.23	0.17	−1.63	1.23	46
BR16	−0.19	−0.70	−0.33	0.88	0.18	0.55	−0.22	−0.06	−0.85	−0.21	−0.28	47
BR6	−0.20	−2.16	−0.51	0.24	2.03	3.60	−1.60	0.78	0.35	3.97	0.37	48
BR17	−0.22	−1.35	1.04	0.96	0.51	−1.47	0.66	−1.34	1.22	0.29	−1.47	49
BR15	−0.23	−1.78	1.13	0.71	0.00	0.37	0.23	1.42	−0.02	−1.37	0.08	50
BR13	−0.25	−1.17	1.11	0.25	−0.29	−0.92	0.78	0.60	−0.78	−1.05	−0.21	51
BR18	−0.26	−0.17	0.31	−1.97	−0.92	0.36	1.22	−0.69	−1.22	0.62	1.22	52
MPR5	−0.31	−1.28	0.36	0.34	0.44	−1.26	0.67	−1.13	1.49	−0.07	0.22	53
PR11	−0.34	−1.19	−0.66	1.11	0.11	0.10	0.36	−0.62	1.08	−1.13	0.15	54
PR5	−0.36	−0.42	−0.60	−0.10	−0.39	−0.51	0.51	−1.40	0.59	−0.01	−0.56	55
BR1	−0.38	−0.28	−0.85	−1.39	0.86	−0.56	−0.34	1.22	−0.85	0.71	−1.12	56
BR14	−0.40	−0.52	−0.60	0.19	−0.83	−0.57	0.40	−0.81	−0.57	−0.56	0.48	57
BR10	−0.41	−1.80	1.24	−0.68	1.24	−0.92	1.19	−1.13	−0.45	−0.78	1.14	58
PR9	−0.44	−0.54	−0.43	0.04	−1.53	0.44	−0.72	−0.83	−0.16	−0.52	0.32	59
BR9	−0.44	−1.04	−0.37	0.39	0.17	−1.09	−0.59	−0.52	0.91	1.39	−1.92	60
PR3	−0.45	−1.03	−0.42	1.10	−1.09	−0.42	−0.11	−0.33	1.00	−0.60	−1.61	61
PR4	−0.46	−1.75	1.22	−1.11	−0.61	−0.25	0.74	0.60	0.67	−0.22	0.45	62
PR1	−0.54	−0.08	−0.71	−0.68	−0.76	−1.76	−1.86	1.19	−2.19	0.18	0.40	63
PR2	−0.54	−0.42	−1.21	−1.23	0.01	−1.62	−0.09	2.31	−0.47	0.07	−1.02	64
PR13	−0.56	−0.76	−1.63	0.38	0.42	0.20	−1.26	−2.05	1.26	−0.03	−0.47	65
BR12	−0.62	−1.11	−0.21	0.00	−1.08	−0.88	−0.79	0.53	−0.42	−0.96	0.07	66
PR10	−0.64	−1.06	−0.52	0.28	−1.04	0.08	−1.35	−0.50	−0.18	−0.96	−0.36	67
PR12	−0.66	−0.85	−2.14	0.47	0.12	−0.16	−0.99	−0.89	0.84	0.46	−1.15	68
BR2	−0.74	−0.99	0.08	−1.58	−0.76	−0.84	−1.68	−0.44	−0.66	1.09	−0.10	69
YR1	−0.99	−0.85	−2.06	−2.24	−0.43	0.15	−0.37	−0.98	−0.16	−0.70	1.23	70
GR1	−1.18	−1.09	−2.60	−2.60	0.18	−0.57	0.30	−0.10	−0.53	−1.25	0.61	71

### Validation of phenotypic characteristics via hierarchical clustering analysis

To verify the reliability of the PCA results, we performed an HCA. We employed the median linkage method for clustering, using Euclidean distance as the similarity measure. Quality indicators were normalized using column-wise Min-Max standardization. As shown in Fig 4, the dendrogram clearly categorized the samples into two major clusters (Cluster I and Cluster II), exhibiting a clustering pattern that was highly consistent with the PCA results.

**Fig 4:**
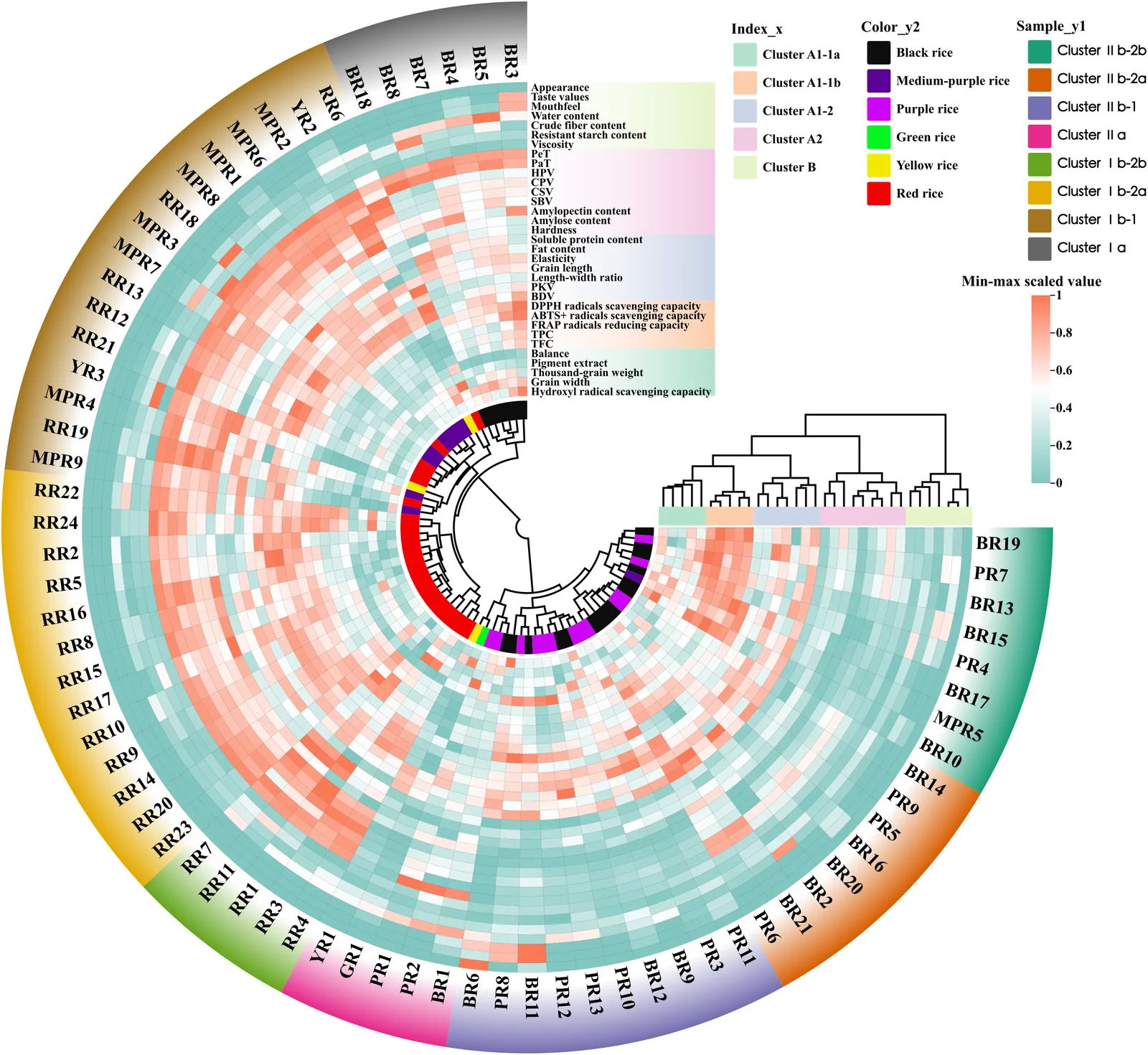
Clustering heatmap of colored rice.

Cluster I comprised the top 40 samples based on PCA ranking, predominantly consisting of red rice and medium-purple rice cultivars. This group displayed significantly higher values for pasting properties (Cluster A2, including hardness, amylose/amylopectin content, SBV, CSV, CPV, HPV, PaT, and PeT), confirming the superior gelatinization characteristics of these cultivars. Within this cluster, Cluster Ia, containing six black rice cultivars, showed notably high PaT and PeT values. Cluster Ib-1 was characterized by slender grain shapes and high fat and soluble protein contents, despite lower antioxidant activity. Cluster Ib-2 consisted exclusively of red rice cultivars, indicating high phenotypic homogeneity.

Cluster II included the remaining 31 samples, mainly composed of black rice and purple rice cultivars, which generally showed low expression levels for pasting properties. Cluster IIa exhibited low values across pasting, nutritional, and antioxidant indicators, consistent with their lower PCA comprehensive scores. Conversely, Cluster IIb demonstrated marked heterogeneity, with Cluster IIb-2b showing distinctively high antioxidant capacity. By integrating HCA and PCA findings, we confirm that pasting properties and antioxidant capacity are the core drivers of phenotypic divergence in colored rice, further validating the robustness of our classification model.

### Analysis of pigment metabolites

To characterize pigment-associated metabolites in different colored rice varieties, non-targeted metabolomic analysis was conducted on pigment extracts from 20 rice samples using UHPLC-MS/MS. A total of 37,204 peaks were detected in both negative and positive ion modes. Following filtering for deviation, missing value imputation, and data standardization, 23,620 peaks were retained for subsequent analysis. Among these, 818 metabolites were identified through MS matching, with 740 metabolites detected in black rice, 712 in red rice, 724 in medium-purple rice, 710 in purple rice, 709 in yellow rice, and 651 in green rice (Fig 5, S7 Table). Notably, 539 metabolites were common across all rice accessions, whereas 279 were identified as differentially accumulated metabolites. These findings align with previous research by Zhang et al. [43], who identified 732 metabolites (including alkaloids, amino acids and derivatives, flavonoids, lignans and coumarins, lipids, nucleotides and derivatives, organic acids, and phenolic acids) from black, red, glutinous, and white rice using UPLC-MS/MS. Their study reported 726 metabolites in black rice, alongside 703, 695, and 682 in red, glutinous, and white rice, respectively. Collectively, these findings indicate that the pigment extracts of colored rice are predominantly composed of flavonoids, alkaloids, and phenolic acids.

**Fig 5:**
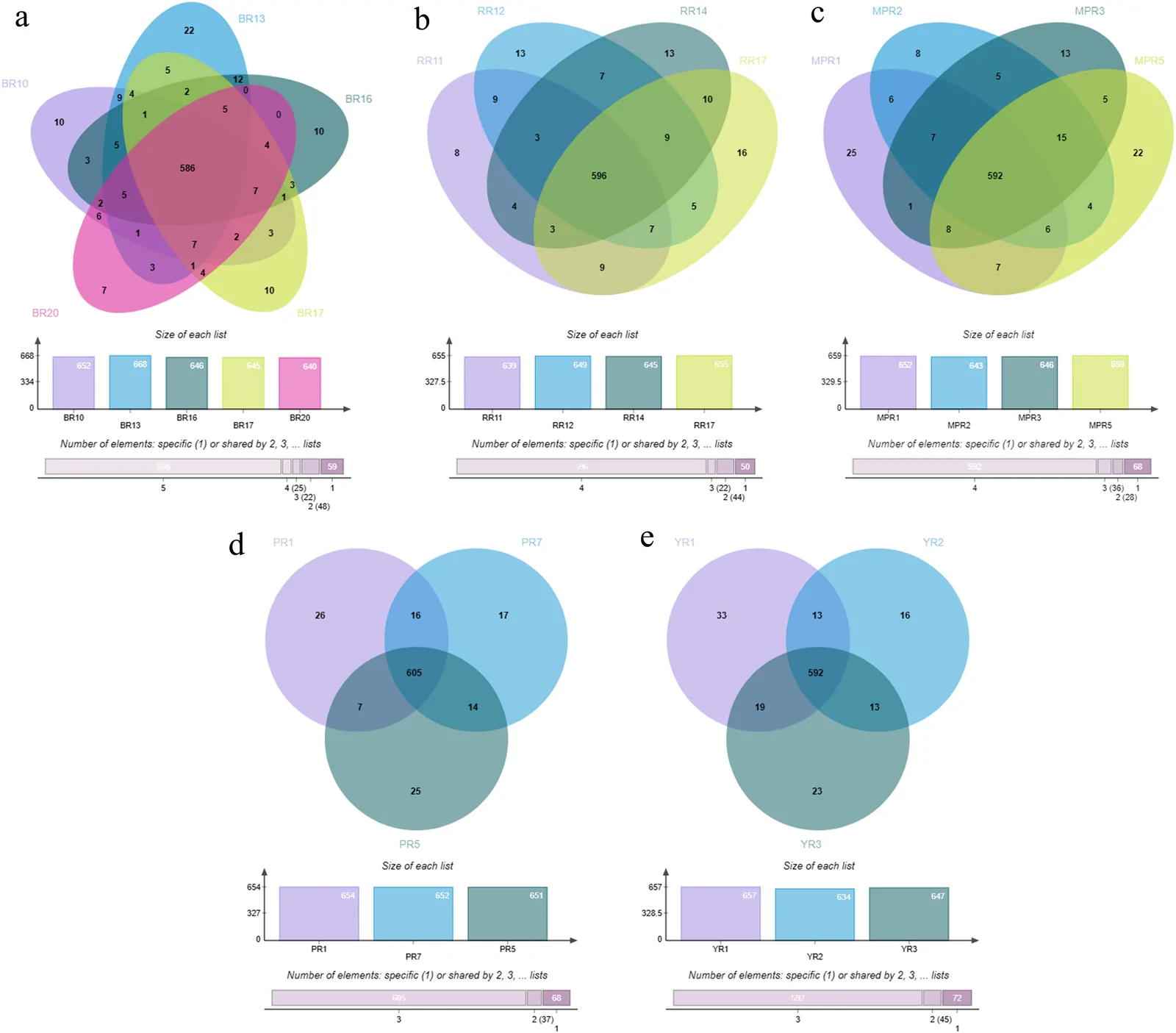
Composition of colored rice pigment extract. Note: Composition of black rice pigment extract (a); Composition of red rice pigment extract (b); Composition of the medium-purple rice pigment extract of rice(c); Composition of purple rice pigment extract (d); Composition of yellow rice pigment extract (e).

Targeted analysis of the metabolomic profiles led to the identification of 15 distinct anthocyanins across the 20 colored rice samples. Of these, 11 anthocyanins were commonly expressed across all samples, while four exhibited differential accumulation. For instance, cyanidin-3-galactoside was specifically detected in black and purple rice accessions but was entirely absent in red and yellow rice, highlighting its role as a distinguishing pigment component for the darker varieties. Furthermore, cyanidin-3-diglucoside-5-glucoside was exclusively found in the BR10 black rice sample, serving as a potential accession-specific biomarker. Regarding procyanidins, three forms were identified: procyanidin C1, B1, and B2. Procyanidin C1 was ubiquitously distributed across all 20 samples. Conversely, procyanidin B2 was restricted to black, red, and purple rice, and procyanidin B1 was exclusively identified in red rice accessions (Table 3).

**Table 3:** Comparison of anthocyanins in 20 pigment extracts.

MS2 name	mz	BR10	BR13	BR16	BR17	BR20	RR11	RR12	RR14	RR17	MPR1	MPR2	MPR3	MPR5	PR5	PR7	PR1	YR3	YR1	YR2	GR1
Malvidin 3-glucoside	493.1343	+	+	+	+	+	+	+	+	+	+	+	+	+	+	+	+	+	+	+	+
Delphinidin 3-glucoside	464.0928	+	+	+	+	+	+	+	+	+	+	+	+	+	+	+	+	+	+	+	+
Isopeonidin 3-galactoside	464.1276	+	+	+	+	+	+	+	+	+	+	+	+	+	+	+	+	+	+	+	+
Tectorigenin 7-O-xylosyl glucoside	595.1641	+	+	+	+	+	+	+	+	+	+	+	+	+	+	+	+	+	+	+	+
4’-O-Methyldelphinidin 3-O-beta-D-glucoside	480.1211	+	+	+	+	+	+	+	+	+	+	+	+	+	+	+	+	+	+	+	+
Delphinidin 3-rutinoside	611.1604	+	+	+	+	+	+	+	+	+	+	+	+	+	+	+	+	+	+	+	+
Cyanidin 3-sambubioside 5-glucoside	743.2034	+	+	+	+	+	+	+	+	+	+	+	+	+	+	+	+	+	+	+	+
Procyanidin C1	865.198	+	+	+	+	+	+	+	+	+	+	+	+	+	+	+	+	+	+	+	+
Cyanidin 3-glucoside	447.0938	+	+	+	+	+	+	+	+	+	+	+	+	+	+	+	+	+	+	+	+
Cyanidin 3-rutinoside	596.1688	+	+	+	+	+	+	+	+	+	+	+	+	+	+	+	+	+	+	+	+
Cyanidin 3-glucogalactoside	612.1647	+	+	+	+	+	+	+	+	+	+	+	+	+	+	+	+	+	+	+	+
Cyanidin 3-(6”-acetyl-galactoside)	491.1193	+	+	+	+	+	+	+	+	+	+	+	+	+	+	+	+	+	+	+	+
Cyanidin 3-galactoside	450.1123	+	+	+	+	+	+	–	–	+	+	+	+	+	+	+	+	–	–	–	+
Cyanidin 3-diglucoside 5-glucoside	774.2147	+	–	–	–	–	–	–	–	–	–	–	–	–	–	–	–	–	–	–	–
Procyanidin B2	577.1357	+	+	+	+	+	+	+	+	+	+	+	+	+	+	+	+	+	+	–	–
Procyanidin B1	577.1343	–	–	–	–	–	–	+	+	+	–	–	–	–	–	–	–	–	–	–	–
Cyanidin 3-(2G-glucosylrutinoside)	757.2194	+	+	+	+	–	–	+	+	+	+	+	+	+	+	+	+	–	–	+	+
Cyanidin 3-O-[b-D-xylopyranosyl-(1- > 2)-[b-D-glucopyranosyl-(1- > 6)]-b-D-galactopyranoside]	744.2069	+	+	+	+	+	–	+	–	+	–	–	+	+	+	+	–	+	+	–	+

Note: “+” indicates that the anthocyanin was detected in the pigment extract; “-” indicates that the anthocyanin was not detected in the pigment extract.

Black rice is conventionally characterized by the presence of anthocyanins such as cyanidin-3-glucoside, cyanidin-3-O-rutinoside, peonidin-3-O-glucoside, malvidin-3-O-glucoside, and delphinidin-3-O-glucoside [44–46], with cyanidin-3-glucoside being the most prevalent, followed by peonidin-3-O-glucoside [13,47]. Similarly, red rice is primarily known to contain cyanidin-3-glucoside, malvidin, peonidin, and cyanidin-3-O-glucoside [48–51]. In this study, although peonidin-3-O-glucoside was not detected, other major anthocyanins—including cyanidin-3-glucoside, cyanidin-3-O-rutinoside, delphinidin-3-glucoside, and malvidin-3-O-glucoside—were successfully identified in both black rice (BR) and red rice (RR), corroborating previous reports [52]. Interestingly, cyanidin 3-(6”-acetyl-galactoside) was detected across BR, RR, PR, MPR, and GR, suggesting that it is not a unique marker for black rice as previously proposed. Additionally, the exclusive detection of procyanidin B1 in RR highlights its potential utility as a specific biomarker for red rice accessions.

For purple rice, typical anthocyanins include cyanidin-3-glucoside, peonidin-3-D-glucoside, cyanidin-3,5-diglucoside, cyanidin-3-rutinoside, and peonidin-3-glucoside [53–55]. However. in this study, peonin-3-D-glucoside, peonidin-3-glucoside, and cyanidin-3,5-diglucoside were absent in the MPR samples. This discrepancy may be attributed to their low natural abundance or the specific genetic background of the MPR accessions evaluated. The intermediate pigmentation of MPR is consistent with the phenotypic observations reported by Rahman et al. [56] in hybrids derived from Heugnambyeo (purple rice) and Hwayongbyeo (white rice). Genetically, the medium-purple pericarp phenotype is expressed in the *Pppp* genotype only when the dominant *Pb* gene is co-expressed. Because the *Pp* allele exhibits incomplete dominance over the recessive *pp* allele, the heterozygous *Pppp* genotype accumulates significantly lower levels of cyanidin-3-glucoside compared to the homozygous *PpPp* genotype (which confers a dark purple coloration). This genetic interaction directly results in the intermediate purple pigmentation characteristic of MPR.

## Conclusion

Colored rice holds significant potential in both the food and healthcare sectors; however, the suboptimal eating quality of many accessions remains a primary challenge, necessitating the development of high-quality, palatable cultivars. In this study, we employed PCA to identify the critical dimensions governing quality across diverse colored rice germplasm resources, revealing that pasting properties, TPC, TFC, and antioxidant capacity are the fundamental drivers of phenotypic divergence. The constructed comprehensive evaluation model identified RR24, RR11, and MPR1 as superior germplasm resources, providing a quantitative framework for future breeding programs. Furthermore, UHPLC-MS/MS analysis identified 15 anthocyanins and 3 proanthocyanidins across 20 pigment extracts. Notably, 11 compounds, including cyanidin-3-glucoside, cyanidin-3-rutinoside, cyanidin-3-galactoside, and proanthocyanidin C1, were identified as the predominant bioactive components.

However, the limitations of this study should be explicitly acknowledged. All nutritional, functional, and antioxidant evaluations were strictly conducted in vitro. Consequently, this study does not demonstrate in vivo bioavailability, digestibility, or definitive causal health effects. Future research should integrate in vivo clinical or animal models to validate the physiological relevance and biological health benefits of these identified bioactive compounds. Despite this limitation, these findings provide a robust scientific foundation for the conservation and utilization of colored rice germplasm, offering critical insights for breeding programs aimed at achieving a balanced profile of superior sensory taste and enhanced nutritional benefits.

## Supporting information

S1 Table Experimental material information. (XLSX)

S2 Table Comparison of evaluation indicators for 71 resources of colored rice. (XLSX)

S3 Table KMO test and Bartlett’s test. (XLSX)

S4 Table Cumulative variance contribution rates in PCA. (XLSX)

S5 Table PC loading coefficients. (XLSX)

S6 Table Calculation formulas for scores of the 10 principal components. (XLSX)

S7 Table Comparison of relative contents of metabolites from 20 colored rice pigment extracts. (XLSX)
